# Extrahepatic alveolar echinococcus on multi-slice computed tomography and magnetic resonance imaging

**DOI:** 10.1038/s41598-021-89101-x

**Published:** 2021-04-30

**Authors:** Hui Guo, Wenya Liu, Jian Wang, Yan Xing

**Affiliations:** State Key Laboratory of Pathogenesis, Prevention and Treatment of High Incidence Diseases in Central Asia, Medical Imaging Center, The First Affiliated Hospital of Xinjiang Medical University, Urumqi, 830054 People’s Republic of China

**Keywords:** Diseases, Health care, Medical research

## Abstract

Alveolar echinococcus (AE) is a severe health problem in endemic areas. In recent years, the incidence of this disease in China has been increasing. The study was designed to illustrate the multi-slice computed tomography (MSCT) and magnetic resonance imaging (MRI) features of extrahepatic AE. A cohort of 33 patients who suffered from extrahepatic AE was enrolled consecutively from January 2012 to December 2017. The MSCT and MRI features of extrahepatic AE were recorded and analyzed by experienced radiologists. All cases secondary to hepatic AE, except two primary extrahepatic AE, were found in this study. Locations of extrahepatic AE included 19 (57.6%) lung, 10 (30.3%) adrenal gland, 9 (27.3%) brain, 5 (15.2%) peritoneal cavity, 5 (15.2%) spleen, 4 (12.1%) diaphragm, 3 (9.1%) kidney, 3 (9.1%) retroperitoneal, and 2 (6.1%) vertebra; Involvement of 1 (3.0%) heart, 1 (3.0%) mediastinum, 1 (3.0%) muscle, and 1 (3.0%) pancreas was rare. AE of the lung usually appeared as irregular and scattered nodules with small vacuoles or cavities inside and peripheral distribution. Multiple cerebral nodules with calcification and surrounding edema were the most common features seen in brain AE. Adrenal gland AE presented as plaques containing different sizes of hypodense areas and different amounts of calcification. Injection of contrast medium showed no enhancement of lesions except in the brain. MSCT and MRI are reliable imaging methods for the diagnosis of extrahepatic AE. When one AE patient is clinically confirmed, MSCT scan from the chest to the abdomen should be performed to exclude other organs AE.

## Introduction

Echinococcosis is a near-cosmopolitan zoonosis caused by an adult or larval stages of cestodes belonging to the genus *Echinococcus* (family Taenlldae). The two major species of medical and public health importance are *Echinococcus granulosus* (*E. granulosus*) and *Echinococcus multilocularis* (*E. multilocularis*), which cause cystic echinococcosis (CE) and alveolar echinococcosis (AE) in the liver and other organs^1,2^, respectively. Both CE and AE are serious and severe diseases, the latter especially so, with high fatality rates and poor prognosis if managed incorrectly.

AE is a widespread, prevalent in the northern hemisphere, including Central Europe, Turkey, Russia, Japan, Alaska, North America and China^3^. It is estimated that 18,000 new cases of AE occur globally each year, including 16,400 in China^4,5^. The primary location of the AE, as a general rule, is almost exclusively in the liver in 97% of cases^6^. In human, the larval mass resembles a malignancy in appearance and behavior because it proliferates indefinitely by exogenous budding and invades the surrounding tissues^7,8^. The larva’s capacity for exogenous proliferation enables it to initiate (usually via the bloodstream) the formation of distant metastases in the lung, brain, bone, kidney and other organs^9^. There is some evidence that AE can be spread through lymphatic drainage^10^.

Although secondary affection of visceral organs is possible, extrahepatic AE is highly uncommon. In the literature, some cases of hepatic AE have been reported^3,11–14^. However, extrahepatic AE was just described sporadically^15,16^. The diagnosis of extrahepatic AE is a challenge even for the experienced examiner because of low human prevalence levels. On the basis of pathological results and clinical experience, multi-slice spiral computed tomography (MSCT) and magnetic resonance imaging (MRI) findings of 33 cases suffering from extrahepatic AE were analyzed in this study.

## Methods

### Subjects

This is a retrospective observational study. 131 patients were clinically confirmed for AE between January 2012 to December 2017, and 33 extrahepatic AE initially considered eligible for our research. After various approaches, including surgical pathology (n = 20), needle biopsy (n = 6), and follow-up of clinical treatment (n = 7), extrahepatic AE lesions were verified in this group. All methods were carried out in accordance with relevant guidelines and regulations. Informed consent was obtained from all patients. The study was approved by the First Affiliated Hospital of Xinjiang Medical University research ethics committee.

### Inclusion and exclusion criteria

Inclusion criteria were as follows: (a) CT and/or MRI were performed in all cases, (b) diagnosis of extrahepatic AE was based on the pathology and clinical follow-up, (c) all cases were more than 18 years.

### Equipments and methods

All examinations were done using MSCT units. Among them 33 patients, 29 were evaluated with 64-slice spiral CT (LightSpeed VCT; GE Healthcare, Milwaukee, WI, USA) and the remaining 4 were examined with 16-slice CT (HiSpeed Advantage CT; GE Healthcare, Milwaukee, WI, USA). After plain CT scanning for the suspected region, a bolus of 60–80 mL of nonionic contrast medium (Ultravist 300, Bayer Schering Pharma, Berlin, Germany) was injected into an antecubital vein at a flow rate of 3.5–5.0 mL/s. Images were acquired with a slice thickness of 5.0–10.0 mm, and 1.25 mm thickness reconstruction imaging was obtained.

MRI examinations were done for 18 patients with the Signa Excite Xl Twin Speed 1.5 T system (8 cases) and Signa Excite HD 3.0 T system (10 cases) (GE Healthcare, Milwaukee, WI, USA) with an 8-channel torso-array coil and/or head coil. Post contrast-enhanced images were obtained after injection of gadopentetate dimeglumine (Gd) 0.2 mmol/kg (Magnevist; Schering, Berlin, Germany) into an antecubital vein at a rate of 1.5–3.0 mL/s.

All CT and MRI images were analyzed and interpreted by two experienced radiologists who were unaware of the pathological findings. Discrepancies in their interpretations were resolved by consensus.

### Statistical analysis

Statistical analysis was performed using the software SPSS version 17.00 (SPSS Inc. Chicago, IL, USA). Quantitative variables were expressed as mean ± SD. P values less than 0.05 were regarded as statistically significant.

## Results

### Demographic characteristics

Demographic characteristics of patients with extrahepatic AE were shown in Table 1. In this study, the incidence of extrahepatic AE was 25.2%, and 33 patients (18 men and 15 women, age range 21–74 years (average 40.67 ± 12.62 years) were suffering from extrahepatic AE rather than hepatic AE. In this group, 31 patients were inhabitants of Xinjiang from birth and two patients were from Kazakhstan. The patients were from multiple ethnic groups, including 19 (57.6%) Han, 8 (24.2%) Kazak, 4 (12.1%) Hui and 2 (6.1%) Tibetan. The most common symptoms were 10 (30.3%) abdominal pain and distention, followed by 8 (24.2%) headache, and 6 (18.2%) masses in the upper abdomen. 31 patients had both hepatic and extrahepatic AE.

**Table 1:** The demographic characteristics and the distribution of AE lesions of 33 cases.

No	Age	Sex	Nation	Clinical symptoms	Location	Treatment	Follow-up time
1	42	F	Hui	Headache	Brain	Albendazole + radical resection	2Y
2	48	F	Han	Abdominal pain	Liver, heart	Albendazole + radical resection	7Y
3	37	M	Kazak	Headache	Live, brain	Albendazole + radical resection	2Y
4	43	F	Hui	Headache	Live, lung, brain	Albendazole	3Y
5	33	F	Han	Headache	Liver, diaphragm	Liver transplantation + albendazole	2Y
6	43	F	Kazak	Headache	Live, lung, brain	Albendazole	1Y
7	51	M	Hui	Distention	Liver, diaphragm	Albendazole + radical resection	3Y
8	21	F	Kazak	Abdominal pain	Live, adrenal gland	Albendazole + radical resection	2Y
9	35	M	Han	Distention	Live, adrenal gland	Albendazole	3Y
10	42	M	Kazak	Mass	Live, adrenal gland	Albendazole + radical resection	4Y
11	43	M	Han	Distention	Live, lung	Liver transplantation + albendazole	2Y
12	47	M	Kazak	Abdominal pain	Live, lung	Albendazole + radical resection	3Y
13	37	F	Han	Distention	Live, lung	Albendazole + radical resection	2Y
14	38	F	Han	Abdominal pain	Live, lung	Albendazole	3Y
15	42	F	Han	Abdominal pain	Live, lung	Albendazole + radical resection	2Y
16	27	F	Tibetan	Abdominal pain	Live, lung	Albendazole + radical resection	1Y
17	31	F	Han	Abdominal pain	Live, lung	Liver transplantation + albendazole	3Y
18	42	F	Kazak	Abdominal pain	Lung	Albendazole + radical resection	2Y
19	73	M	Han	Abdominal pain	Live, lung	Albendazole	3Y
20	30	M	Han	Distention	Live, lung, diaphragm	Albendazole + radical resection	4Y
21	47	M	Hui	Mass	Live, lung, adrenal gland	Albendazole + radical resection	1Y
22	56	F	Han	Distention	Live, spleen, peritoneal cavity	Albendazole + palliative management	2Y
23	41	M	Han	Mass	Live, lung, kidney, adrenal gland	Albendazole	7Y
24	40	M	Han	Headache	Liver, adrenal gland, kidney, brain	Albendazole	2Y
25	37	M	Tibetan	Distention	Live,lung, diaphragm, adrenal gland	Albendazole	3Y
26	41	F	Han	Distention	Liver, peritoneal cavity, pancreas	Albendazole	2Y
27	62	F	Han	Headache	Live, lung, adrenal gland, brain	Albendazole	1Y
28	21	M	Kazak	Abdominal pain	Live, lung, adrenal gland, brain	Albendazole + radical resection	3Y
29	25	M	Han	Mass	Live, lung, spleen, kidney, brain, vertebra, muscle	Albendazole + palliative management	2Y
30	36	M	Han	Headache	Liver, spleen, peritoneal cavity, lung, mediastium, brain	Albendazole	3Y
31	31	M	Han	Mass, distention	Live, spleen, peritoneal cavity, retroperitoneal	Albendazole + radical resection	4Y
32	26	F	Han	Distention	Live, peritoneal cavity, retroperitoneal	Albendazole + radical resection	1Y
33	74	M	Kazak	Mass	Spleen, adrenal gland, vertebra, retroperitoneal	Albendazole	2Y

*M* Male, *F* Female, *Y* Year.

### Distribution of the extrahepatic AE

The distribution of the extrahepatic AE were shown in Table 1. In this study, 31 patients had both hepatic and extrahepatic AE, and 2 cases were primary extrahepatic AE (brain and lung). The organs involved included 19 (57.6%) lung, 10 (30.3%) adrenal gland, 9 (27.3%) brain, 5 (15.2%) peritoneal cavity, 5 (15.2%) spleen, 4 (12.1%) diaphragm, 3 (9.1%) kidney, 3 (9.1%) retroperitoneal, and 2 (6.1%) vertebra; Involvement of 1 (3.0%) heart, 1 (3.0%) mediastium, 1 (3.0%) muscle, and 1 (3.0%) pancreas was rare.

### MSCT and MR imaging findings of the hepatic AE

Thirty-one cases underwent CT scan, and 16 cases did MRI scan; The minimum lesion is 11 × 13 × 19 mm^3^, and the maximum was 207 × 162 × 195 mm^3^.

On plain CT scan, a total of 32 lesions presented as massive low-density masses with uneven density and blurred boundaries. The CT value of the focal parenchyma was 37–74 Hu. Scattered small calcifications clustered around the parenchyma or liquefied necrotic area at the edge of the lesion in 26 cases; scattered lesions were observed in 19 cases or cluster of small vesicles with a diameter of 1 to 15 mm, which are more likely to be shown near the edge of the lesion. Contrast-enhanced scan showed no significant enhancement in the lesion parenchyma, but enhancement in the surrounding liver parenchyma made the lesion boundary clear.

On plain MRI, all Lesions exhibited hypo- or isointense in a T1-weighted image and heterogeneous hyperintense in a T2-weighted image. No enhancement of lesions was seen after a contrast enhancement scan, but the alveoli-like pattern inside the lesions was seen more clearly.

### MSCT and MR imaging findings of the extrahepatic AE

#### AE of the lung

**Figure 1:**
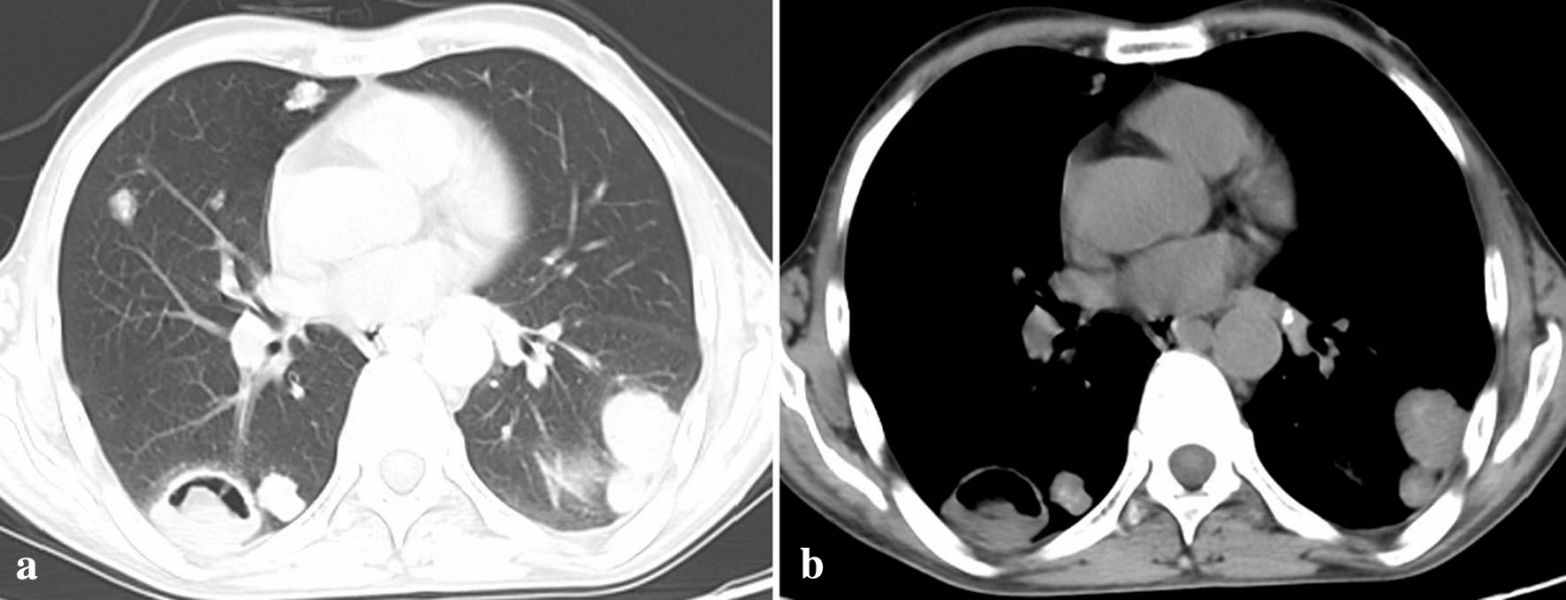
Multiple irregular nodules with different morphology in MSCT lung setting image (**a**) and mediastinum setting image (**b**).

Multiple AE lesions were found in 15 (78.9%) cases and a single AE lesion in 4 (21.1%) cases. Bilateral lung was found in 11 (57.9%) cases. Typically, AE lesions appeared as multiple nodules (14 cases) with peripheral locations (18 cases). Those nodules showed infiltrating contours (15 cases) with heterogeneous density (16 cases). Calcification was string-like or patch-like within the lesions in 16 cases. Small vacuoles and eccentric cavities were found inside the lesions in 12 cases. Multiple nodules with different morphology were seen in 9 cases (Fig. 1). One case presented multiple hypodense masses without calcification, leading to a misinterpretation of the MSCT images.

#### AE of the adrenal gland

**Figure 2:**
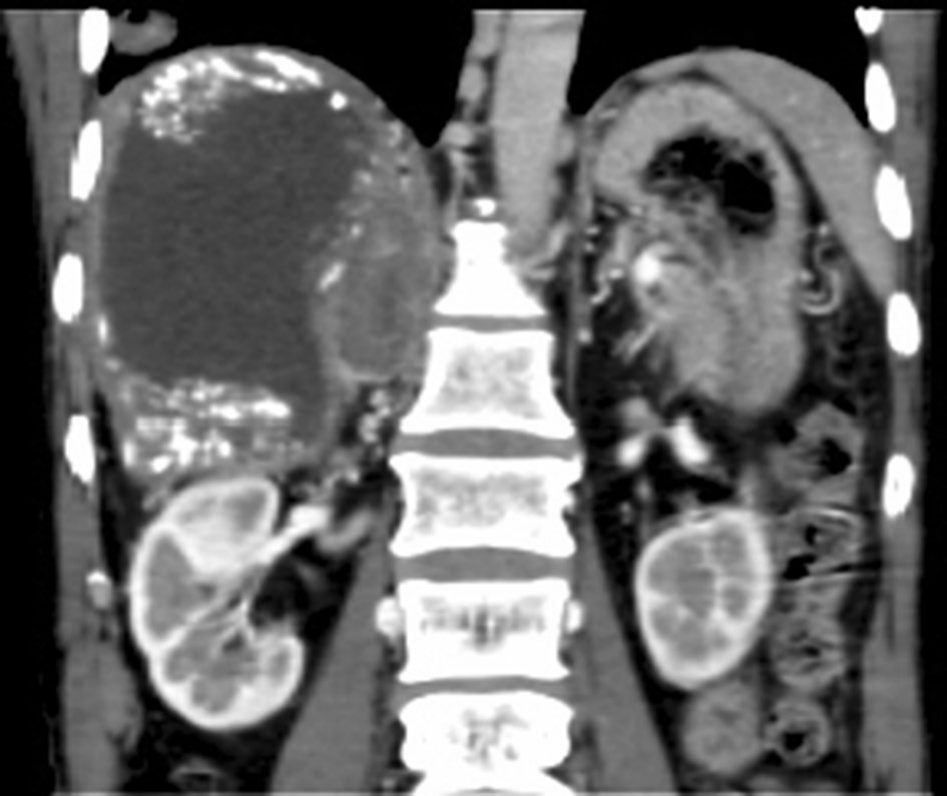
Hepatic AE involved the right adrenal gland. A mixed density mass in the right adrenal gland was visible in the coronal CT image.

AE lesions were located in the adrenal gland in 10 cases. All lesions were recognized as a single mass in the right adrenal gland. Five cases seemed to be directly invaded by AE lesions in the right lobe of liver. However, they were associated with 2 lung metastases, 2 lung and brain metastases, 1 kidney and brain metastases; 5 cases were metastatic lesions from the liver lesion. On MSCT scan, lesions were slightly hypodense with different degrees of necrosis in 4 cases, and lesions were mixed-density masses with various calcification degrees in 6 cases (Fig. 2). Six cases underwent MRI scan; all masses exhibited hypo- or isointense in a T1-weighted image and heterogeneous hyperintense in a T2-weighted image. No enhancement of lesions was seen after a contrast enhancement scan, but the alveoli-like pattern inside the lesions was seen more clearly.

#### AE of the brain

**Figure 3:**
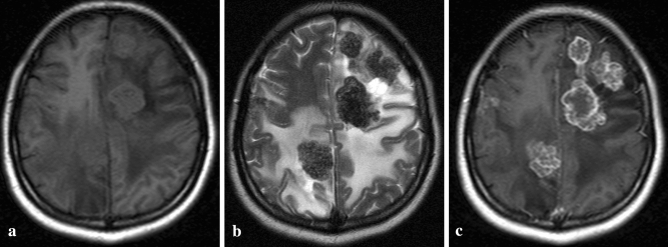
Multiple cerebral lesions were isointense in the T1-weighted image (**a**) and heterogeneously hypointense in the T2-weighted image (**b**) with surrounding perilesional edema. All lesions were enhanced peripherally following injection of contrast (**c**).

Multiple lesions were found in all 8 cases and the number of lesions ranged from 2 to 7; the single lesion was seen in one patient. The cerebrum was involved in 5 cases, and both cerebrum and cerebellum were involved in 3 cases. AE lesions appeared in the MSCT scan as hypodense nodules in 7 cases and as isodense nodules in 1 case. All lesions were enhanced in the periphery after the injection of contrast. Five cases also underwent an MRI scan; all lesions were isointense on T1-weighted images and were heterogeneously hypointense on T2-weighted images. Cerebral lesions were enhanced peripherally after the injection of contrast (Fig. 3). Three more nodules were found in enhanced MR images than those found in the MSCT images. Edema of different degrees was detected within the lesion in all cases, accompanied by the ventricle system’s displacement in 4 cases.

**Figure 4:**
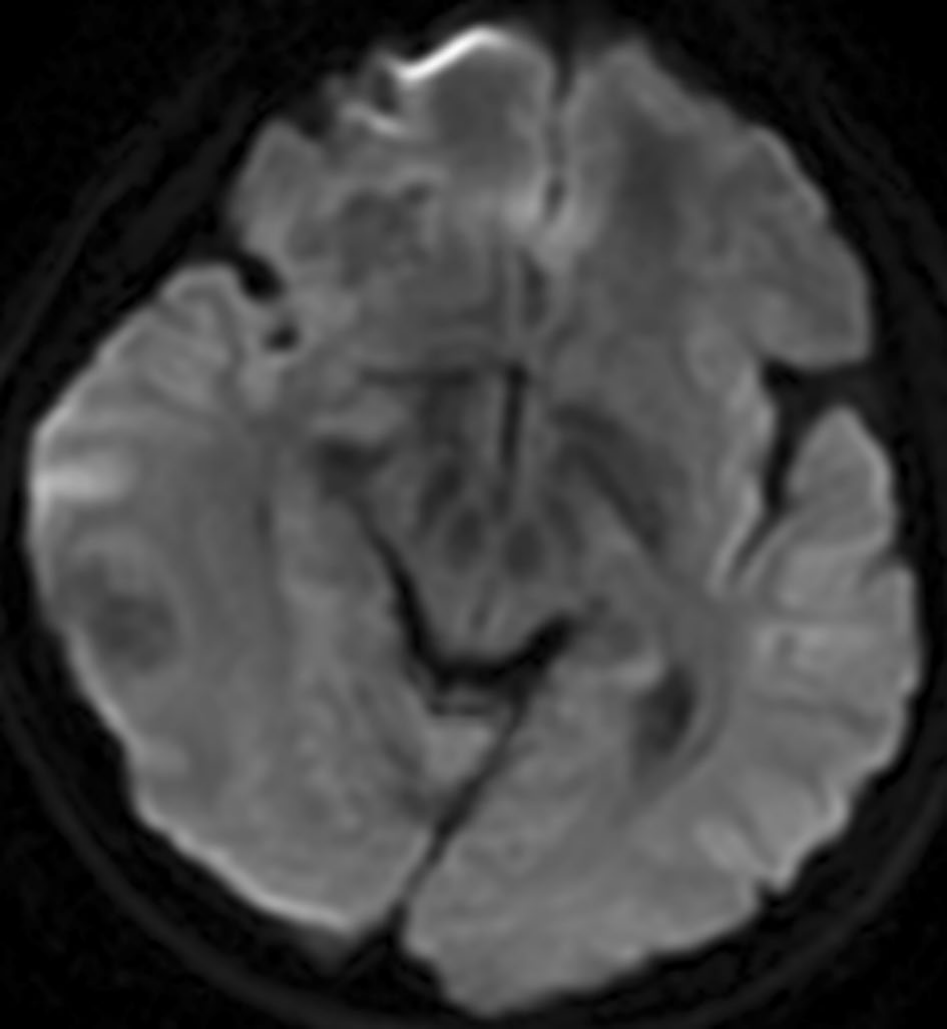
Two lesions were hyperintense signal on a axial MR DWI image.

MR DWI examination was performed in 5 patients. DWI showed that the free diffusion characteristics of all cases were low signal intensity on high b value DWI image and high signal on ADC (Fig. 4).

#### AE of the spleen

**Figure 5:**
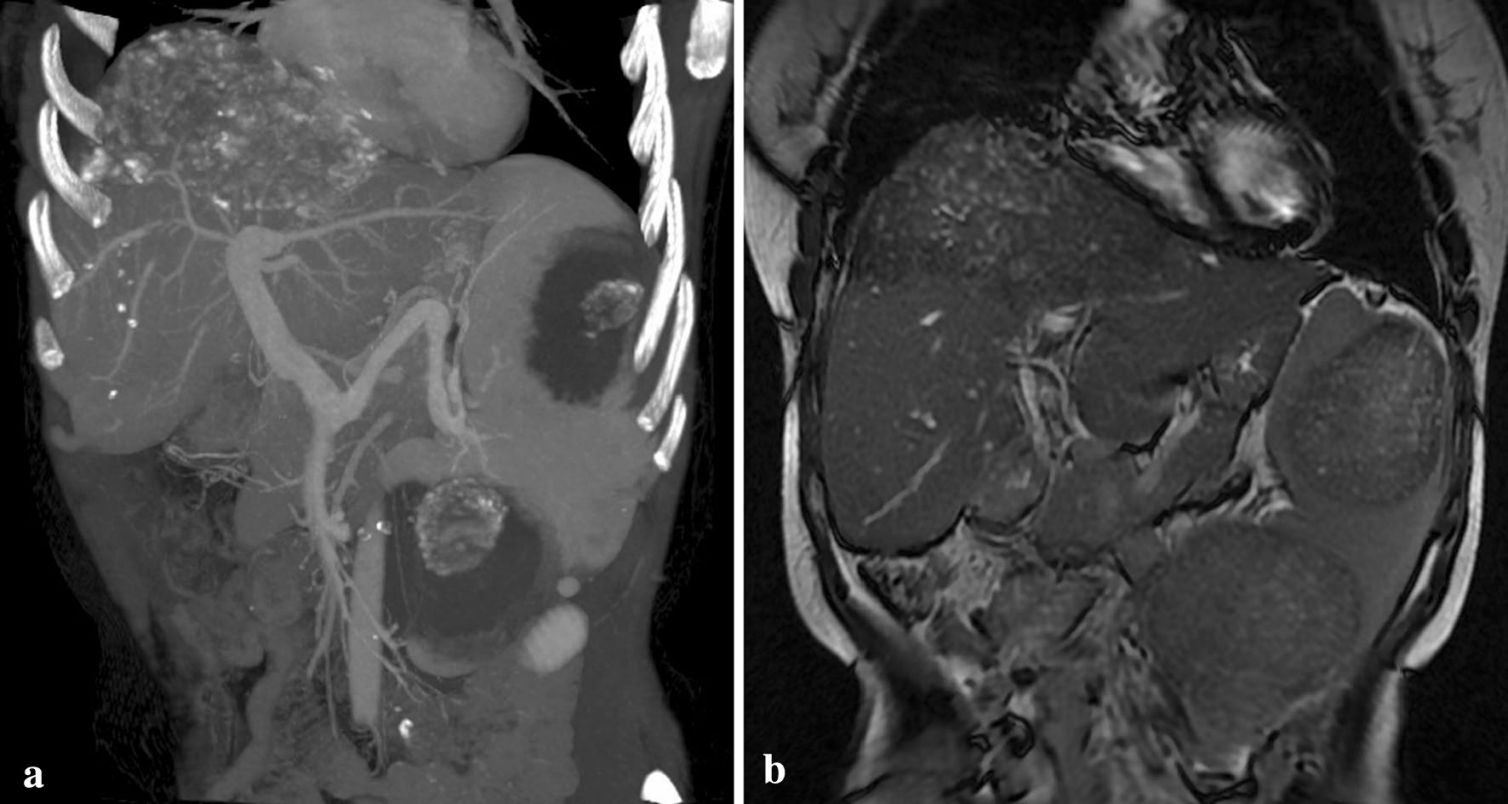
Two lesions were low density with nodular calcification in a coronary MSCT image (**a**) and heterogeneous intensity in a coronary T2-weighted MR image (**b**). This case also had hepatic AE.

AE lesions were located in the spleen in 5 cases; altogether, 6 lesions with sizes ranging from 1.8 to 6.5 cm were found. Four lesions showed hypodensity in MSCT images. One case showed nodular calcification within 2 lesions (Fig. 5). All lesions detected by MRI were hypointense in T1-weighted images and were homogeneously hyperintense in T2 -weighted images.

Vertebral involvement was found in 2 cases. One case had metastatic lesions of the liver and the other case was disseminated from the spleen AE. Heterogeneous osteolysis and irregular bone destruction were seen in both cases. In the soft tissues, MSCT revealed heterogeneous masses with irregularly thickened septations and scattered calcification and the MRI scan showed multi-vesicular morphology more clearly.

Heart involvement was found in 1 case. The lesion located in the myocardium of the left ventricle showed a mixed density mass with several irregular or ring-like calcifications in the periphery and multiple vacuoles dispersed inside. The mass compressed the chamber of the left ventricle and extended into the pericardial cavity. The mass showed no enhancement after injection of contrast.

Difform nodules or masses were seen in the AE lesions of the peritoneal cavity (5cases), diaphragm (4 cases), kidney (3 cases), retroperitoneal (3 cases), pancreas (1 case), mediastinum (1 case) and muscle (1 case). All of them were not contiguous with hepatic AE lesion. Calcification and necrosis were the common features of these lesions.

### Treatment and follow-up of the extrahepatic AE patients

Treatment and follow-up of patients with extrahepatic AE were shown in Table 1. Treatment and management were provided according to the location, number and size of the lesions and the general condition of the patients; 16 patients underwent radical resection following albendazole therapy, 3 accepted liver transplantation plus albendazole therapy, 2 received albendazole therapy and palliative management and 12 received albendazole therapy only. These patients’ medical history was traced back for 1–7 years (average 2.64 ± 1.49 years). There were 23 cases of improvement, 7 cases of recurrence, and 3 cases of death.

## Discussion

AE is a serious health problem in endemic areas. Human extrahepatic AE disease is even more rare, even in areas where hydatid disease is endemic. To our knowledge, it is the most significant number of cases with extrahepatic AE in worldwide. The incidence of extrahepatic AE was 25.2% in xinjiang of China.

The mean age of the patients was 41 years (range 21–74 years) and 54.5% were men. Tilmann Graeter had also reported that the mean age of the patients was 50 years (range 21–74 years) and 56.2% were men^12^. Tilmann Graeter’s study was a multicenter study, including German, French, and Chinese patients, but our study is a single-center study of larger cases.

The symptoms and clinical signs depend on the local anatomical structures and/or organs affected. In the current study, the most common symptoms were 10 (30.3%) abdominal pain and distention, followed by 8 (24.2%) headache, and 6 (18.2%) masses in the upper abdomen. One of the patients had the symptoms of distention and mass. 31 patients had both hepatic and extrahepatic AE. Therefore, the clinical presentation of extrahepatic AE is highly variable^17^. Owing to its late onset and variety of clinical features, a postponed diagnosis is fairly common.

Ultrasonography is a basic diagnostic imaging tool for general investigation and clinical course in AE. Complications of bile ducts or vessels of the liver can also be detected by ultrasonography^18^. Nevertheless, ultrasonography is inadequate for evaluating extrahepatic AE because of the variety of organs affected, such as lung and brain. Both CT and MRI can indicate the location and imaging characteristics of extrahepatic AE, and reveal the relationship with adjacent structures. Some pathologic features of AE lesions have been found by CT and MRI, and these two modalities usually complement each other^19^.

Among our study cases, the lung was the most frequent extrahepatic organ (57.6%) with AE, followed by the adrenal gland (30.3%) and brain (27.3%). Lung AE lesions usually appeared as irregular and scattered nodules with small vacuoles or cavities inside and peripheral location. The most lesions of multiple nodules were 78.9%, and bilateral lung was seen in 57.9% of cases. AE lesions in the right adrenal gland usually present as a solitary mass containing different amounts of calcification. Although there are reports that the bilateral/left adrenal gland can be affected by AE^20^, all lesions in this study involved only the right adrenal gland. Calcification (8 cases) and surrounding edema (8 cases) were common features of brain AE. Most lesions showed no enhancement by contrast, except brain AE lesions. It has been suggested that the enhancement of the lesions is due to disruption of the blood–brain barrier and is accompanied by a surrounding inflammatory reaction^21^.

Imaging plays an essential role in identifying extrahepatic lesions. Localization within the extrahepatic AE may be based on CT or MRI. In this study, altogether 25 organs of 18 patients, performed MSCT and MRI, very good was seen to detect size, border and size of extrahepatic AE lesions. A moderate agreement was seen for detecting necrosis inside the lesions. In the center of the lesion, liquid necrosis forms irregular cavities containing gelatinoid substances, resulting in low density on CT image and high intensity in the T2-weighted image in the lesion center. A fair agreement was seen for detecting calcification inside the lesions. The necrosis and degeneration of the lesion cause calcification of various degrees and morphology, such as plaques and ring-like calcifications formed in the periphery of the vacuoles, which is characteristic of this disease. Calcification manifested as hyperdensity was seen clearly on MSCT images. T2 shortening of some lesions in MR images might be caused by calcification. We speculated the calcification of pulmonary AE lesions may be related to the strong encapsulation of the necrotic worm. When the hydatid body of AE reaches a alveolus through the blood and stays in it, the inflammatory medium rapidly envelops it and forms AE micro nodule. In the AE micro nodule growing rapidly, the necrotic hydatid body which is caused by insufficient blood supply is encapsulated by inflammatory mediators again. After a series of chemical evolution, calcification is formed.

In our study, DWI showed that the free diffusion characteristics of all cases were low signal intensity on high b value DWI image. DWI can reflect the tissue structure at the molecular level. Under the pathophysiological state, the function of water molecules changes, and then reflects the early morphological and physiological changes related to the change of tissue water content^22^.

As extrahepatic hydatid involves multiple organs and CT scan is fast and inexpensive, and diagnosing the image of AE is comparable to MRI. A standard of care should be established for extrahepatic AE disease; A study recommend a MSCT scan from the head to the pelvis^15^.

Invasion of the diaphragm, peritoneum, mesentery, spleen, pancreas, adrenal glands, kidneys, gallbladder, abdominal wall, and stomach have been reported^23^. In the current study, all lesions of the right adrenal gland were recognized as a single mass. Five cases seemed to be directly invaded by AE lesions in the right lobe of liver, and other 5 cases were metastatic lesions from the liver lesion. 5 cases of abdominal AE lesions, 4 diaphragm, 3 kidney, 3 retroperitoneum, 1 pancreas, 1 muscle were not adjacent to hepatic AE lesions, which were all caused by metastasis. CT and MRI showed nodules or masses, and calcification and necrosis were the common features of these lesions.

The spectrum of differential diagnosis of extrahepatic AE is broad and includes mainly metastases, tuberculomas and fungal infections^24^. Radiological diagnosis of extrahepatic AE can be difficult even when the presence of a primary hepatic lesion is known. Therefore, geographical prevalence, clinical history of hepatic involvement and serological tests are useful for reaching a preliminary diagnosis, but histopathological examination is necessary for a definite diagnosis.

There are limitations to this study that should be addressed. Extrahepatic AE is rare and there were too few cases in this study for some sites to identify the characteristics in MSCT and MR images. This was a retrospective study and the examination parameters of MSCT and MRI were not uniform. A follow-up study is underway.

In conclusion, when one AE patient is clinically confirmed, MSCT scan from the chest to the abdomen should be performed to exclude other organs AE. Extrahepatic AE can be located in many organs or tissues. Extrahepatic AE has certain imaging features on MSCT and MRI. Hence, familiarity with the imaging findings of extrahepatic AE may help make an accurate diagnosis and prevent potential complications.

## Abbreviations

CT: Computed tomography
MSCT: Multi-slice computed tomography
MRI: Magnetic resonance imaging
AE: Alveolar echinococcus
CE: Cystic echinococcosis
Gd: Gadopentetate dimeglumine
SD: Standard deviation
